# A comprehensive review of current insights into the virulence factors of SARS-CoV-2

**DOI:** 10.1128/jvi.02049-24

**Published:** 2025-01-29

**Authors:** Yi Wang, Bingqing Xia, Zhaobing Gao

**Affiliations:** 1 State Key Laboratory of Drug Research, Shanghai Institute of Materia Medica, Chinese Academy of Sciences 58298 , Shanghai, China; 2 University of Chinese Academy of Sciences 74519 , Beijing, China; Universiteit Gent, Merelbeke, Belgium

**Keywords:** SARS-CoV-2, virulence factors, envelope protein, pathogenicity

## Abstract

The evolution of SARS-CoV-2 pathogenicity has been a major focus of attention. However, the determinants of pathogenicity are still unclear. Various hypotheses have attempted to elucidate the mechanisms underlying the evolution of viral pathogenicity, but a definitive conclusion has yet to be reached. Here, we review the potential impact of all proteins in SARS-CoV-2 on the viral pathogenic process and analyze the effects of their mutations on pathogenicity evolution. We aim to summarize which virus-encoded proteins are crucial in influencing viral pathogenicity, defined as disease severity following infection. Mutations in these key proteins, which are the virulence factors in SARS-CoV-2, may be the driving forces behind the evolution of viral pathogenicity. Mutations in the S protein can impact viral entry and fusogenicity. Mutations in proteins such as NSP2, NSP5, NSP14, and ORF7a can alter the virus’s ability to suppress host protein synthesis and innate immunity. Mutations in NSP3, NSP4, NSP6, N protein, NSP5, and NSP12 may alter viral replication efficiency. The combined effects of mutations in the S protein and NSP6 can significantly reduce viral replication. In addition, various viral proteins, including ORF3a, ORF8, NSP4, Spike protein, N protein, and E protein, directly participate in the inflammatory process. Mutations in these proteins can modulate the levels of inflammation following infection. Collectively, these viral protein mutations can influence SARS-CoV-2 pathogenicity by impacting viral immune evasion, replication capacity, and the level of inflammation mediated by infection. In conclusion, the evolution of SARS-CoV-2 pathogenicity is likely determined by multiple virulence factors.

## INTRODUCTION

Since 2019, SARS-CoV-2 has precipitated an unprecedented global health crisis (1). While many patients infected with the virus exhibit mild to moderate symptoms, including cough, fever, and headache, some progress to severe pneumonia and respiratory failure (1, 2). Severe patients typically experience significant dysfunctional immune responses and cytokine storms, which can progress to acute respiratory distress syndrome (ARDS) (3). The severity of the disease is determined by multiple factors, including individual susceptibility, differences in immunity, and pathogen virulence. As new variants continue to emerge, clinical and experimental results reveal that the pathogenicity of the virus is constantly evolving (4–6). Compared to the wild-type strain, infection with the Alpha and Beta variants results in a higher mortality rate in the K18-hACE2 mouse strain, which is susceptible to SARS-CoV-2 (7). The heightened fusogenicity caused by the P681R mutation in the spike protein of the Delta variant could be a factor contributing to increased severity and unusual symptoms (8). Additionally, the reduced spread of the Omicron variant in lung tissue might be related to its attenuated pathogenicity (9). However, it remains unclear which proteins of SARS-CoV-2 play a decisive role in this process. There is an imperative to understand the internal mechanisms of virus pathogenicity evolution, which is conducive to virus virulence prediction and drug development. Various theories have attempted to explain the pathogenic mechanisms of SARS-CoV-2. Mutations in accessory proteins such as ORF3a and ORF8 can disrupt innate immune signaling, impacting viral pathogenicity (10). The fusogenicity and S1/S2 cleavage efficiency of SARS-CoV-2 may be associated with its pathogenicity (8, 9). Mutations in the Spike protein and NSP6 can reduce replication efficiency in Omicron, thereby weakening its pathogenicity (11). Mutations in the E protein can attenuate its intrinsic toxicity, potentially contributing to reduced pathogenicity (12, 13). Additionally, mutations in NSP4 can inhibit chemokine secretion and reduce host inflammatory responses, thereby attenuating the pathogenicity of the Omicron variant (14). While these studies provide some insights into the pathogenicity changes associated with certain variants, they could not fully explain the evolution of SARS-CoV-2 pathogenicity. For instance, ORF3a is unmutated in both the Alpha and Omicron variants, while the E protein and NSP4 are unmutated in Alpha, Gamma, and Delta variants, suggesting that these proteins do not drive the pathogenicity differences among these variants of concern (VOCs). Mutations in the Spike protein appear closely linked to viral pathogenicity. However, individual Spike protein mutations alone do not replicate the pathogenic profile observed in variant strains (11). Therefore, the pathogenicity of SARS-CoV-2 seems to be the result of multiple virulence factors acting in concert. Systematically understanding the role of SARS-CoV-2 virulence factors and their mutations is essential for elucidating the pathogenic mechanisms. Here, we conduct a thorough review of existing literature to integrate findings from experimental studies, epidemiological data, and clinical research on the subject. We believe that this combined review will allow us to gain deeper insights into the factors driving viral pathogenicity and provide a comprehensive understanding of the mechanisms that differentiate the VOCs in terms of their virulence.

## THE CLASSIFICATION OF SARS-CoV-2 PROTEINS

**Fig 1:**
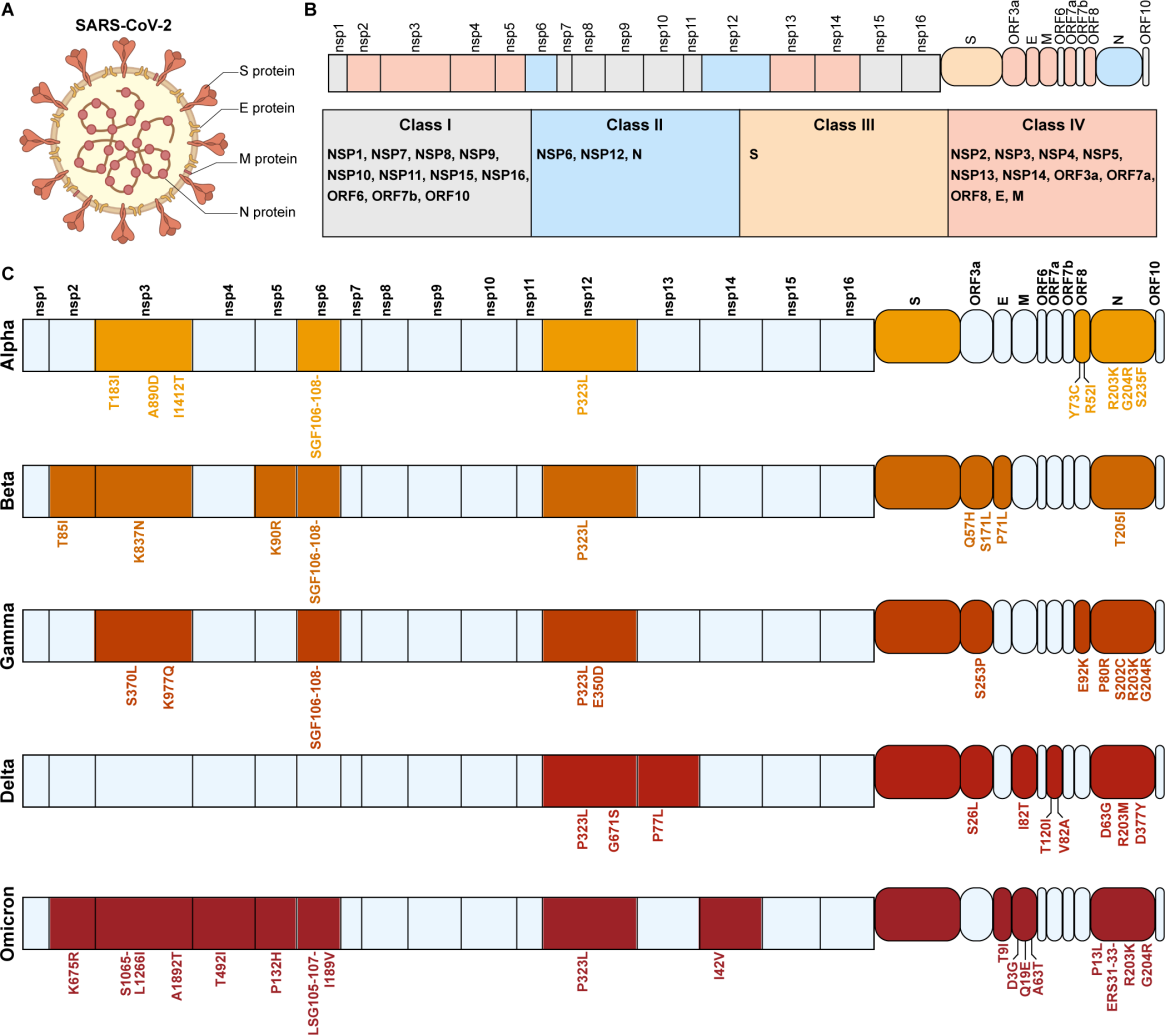
The classification of SARS-CoV-2 proteins based on spontaneous mutation in five VOCs. (**A)** A schematic diagram of SARS-CoV-2. The four structural proteins of the virus are labeled accordingly.** (B)** The genome of SARS-CoV-2 and the classification of its proteins. Proteins within the gray squares represent Class I, with no mutations observed in these proteins across the five VOCs. Light blue squares represent Class II, comprising proteins with conserved mutations across the five VOCs. Orange squares denote Class III, including the Spike protein, which is characterized by a high frequency of mutations. Red-orange squares represent Class IV, containing proteins that are relatively conserved but exhibit mutations in certain VOCs. (**C)** The spontaneous mutations in the five VOCs. Proteins marked in red indicate the presence of mutations within the specific VOC.

SARS-CoV-2 contains 4 structural proteins, 16 nonstructural proteins, and multiple accessory proteins (15, 16) (Fig. 1A and B; Table 1). Mutations in these proteins confer unique transmissibility, immune escape potential, and pathogenicity on emerging variants, which in turn drives the continuation of the COVID-19 pandemic (5, 8–10, 17). We analyzed mutation profiles of viral proteins across the five VOCs via the National Genomics Data Center, identifying the conserved mutations within each VOC (Fig. 1C). Based on the mutations in the five VOCs, all proteins can be roughly divided into four classes (Fig. 1B): Class I includes proteins that did not mutate in any of the five VOCs, such as NSP1, NSP7, NSP8, NSP9, NSP10, NSP11, NSP15, NSP16, ORF6, ORF7b, and ORF10, suggesting that these proteins have a minimal impact on the current changes in virus pathogenicity. Class II includes proteins with mutations that are relatively conserved across the VOCs, such as NSP6, NSP12, and N, indicating that these mutations are beneficial for viral fitness. Class III includes proteins with high-frequency mutations, such as S. Class IV includes relatively conserved proteins but with mutations in some VOCs, such as NSP2, NSP3, NSP4, NSP5, NSP13, NSP14, ORF3a, E, M, ORF7a, and ORF8. Some mutations have been retained in the current Omicron subvariants, becoming the main mutations in the prevalent strains, suggesting they may be related to the pathogenicity of the virus at this stage (13, 14). All four classes of proteins may be involved in the virus’s pathogenic process, but identifying which mutations are the key factors affecting changes in virus pathogenicity is crucial. Here, we review how these proteins influence the pathogenesis of SARS-CoV-2 and analyze their potential impact on the evolution of the virus’s pathogenicity.

**TABLE 1:** Main function of SARS-CoV-2 proteins

Protein	Functions
NSP1	Leader protein, inhibits host protein synthesis and suppresses innate immunity
NSP2	Binds nucleic acids and regulates intracellular signaling pathways
NSP3	PLpro, cleaves the polyproteins
NSP4	Involved in the formation of double-membrane vesicles
NSP5	3CLpro, cleaves the polyproteins
NSP6	Promotes the formation of replication organelles and regulates host cell autophagy
NSP7	Cofactor of RNA-dependent RNA polymerase (RdRP), participates in replication and transcription complex (RTC) formation
NSP8	Cofactor of RdRP, participates in RTC formation
NSP9	RNA-binding protein, participates in RTC formation
NSP10	Cofactor of NSP16, involved in viral RNA methylation
NSP11	Contains only 13 amino acids, with an unknown function
NSP12	RdRP, core component of RTC, catalyzes viral RNA synthesis
NSP13	Helicase, participates in RTC formation
NSP14	3′-to-5′ exonuclease, corrects errors in the RNA synthesis complex
NSP15	EndoRNase, a uridylate-specific endoribonuclease
NSP16	2′-O-ribose methyltransferase, mediates viral RNA methylation
ORF3a	Involved in viral replication and release, mediates inflammation
ORF6	Inhibits innate immunity
ORF7a	Inhibits the IFN signaling pathway
ORF7b	Inhibits the IFN signaling pathway
ORF8	Regulates the host immune response
ORF10	Inhibits innate immunity
S	Recognizes cell receptors and participates in the virus-cell fusion process
E	Forms a viroporin, involved in viral assembly and release, induces ARDS-like damage
M	Determines the shape of the viral envelope, involved in viral particle assembly
N	Compact viral genomic RNA

### Class I (NSP1, NSP7, NSP8, NSP9, NSP10, NSP11, NSP15, NSP16, ORF6, ORF7b, and PRF10)

Although the proteins of Class I do not exhibit mutations in any of the five VOCs, numerous studies suggest that these proteins may play roles in the pathogenic process of the SARS-CoV-2, including inhibiting the host innate immune response, promoting viral replication, and enhancing the release of inflammatory factors.

#### NSP1 (leader protein)

NSP1 is the first translated protein of the ORF1a, consisting of 180 amino acids. NSP1 can inhibit host cell translation and suppress the host’s innate immune response. In 2020, Thoms et al. (18, 19) identified the structure of the NSP1-human 40S ribosomal subunit complex via cryo-electron microscopy, revealing that NSP1 blocks mRNA entry into the translation tunnel, thereby inhibiting RIG-I-dependent innate immune responses, exacerbating viral infection. Additionally, studies found that only full-length NSP1 is fully functional, with multiple mutations abolishing its cytotoxicity, indicating that its function requires coordination between multiple domains (20, 21). However, no NSP1 mutations have been found in the five VOCs, suggesting it is not a key driver in the evolution of viral pathogenicity.

#### NSP7 and NSP8

NSP7 and NSP8 are both cofactors of NSP12, capable of forming a primase complex that plays a crucial role in regulating RNA-dependent RNA polymerase (RdRP) (22). Similarly, NSP7 and NSP8 are key proteins involved in the formation of the replication and transcription complex (RTC), impacting viral replication (23). Additionally, NSP7 and NSP8 have been found to participate in host immune regulation, thereby impacting viral pathogenicity. Studies showed that NSP7 shares homology with the T regulatory cell epitope (Tregitope 289), enabling it to suppress CD4^+^ and CD8^+^ T cell memory responses and evade host immune defenses (24). NSP8 can mediate γδ T cell recognition and play a role in suppressing pathogenic replication (25). Pan et al. (26) reported that the NSP8-S76F mutation increases the virulence of recombinant SARS-CoV-2 in mice.

#### NSP9, NSP15 (EndoRNase), NSP16 (methyltransferase), ORF6, ORF7b, and ORF10

In addition to NSP1, other proteins of Class I play regulatory roles in innate immunity. NSP9 interferes with host cell protein trafficking by binding to the signal recognition particle, affecting the secretion of interferons and cytokines (27). Yuen et al. (28) discovered that NSP15 and ORF6 are potential interferon antagonists, effectively inhibiting IFN expression and IFN-related signaling pathways. NSP15 is a key protein involved in suppressing the host’s innate immunity. The loss-of-function mutations in NSP15 can weaken the virus’s ability to suppress innate immunity, thereby affecting viral replication in cell cultures or primary nasal epithelial air–liquid interface cultures (29). Additionally, NSP15 can prevent the formation of autophagosomes, inhibiting the host cell’s ability to degrade the virus via the autophagy/lysosome pathway (30). ORF6 plays a critical role in evading innate immunity, and its mutations may affect viral immune escape. ORF6 can block the nuclear import of IRF3 and STAT1 to suppress IFN expression (31). Addetia et al. (32) found that ORF6 interacts with the mRNA export factors Rae1 and Nup98, blocking the nuclear pore complex, which in turn inhibits nuclear import and export and renders host cells incapable of responding to SARS-CoV-2 infection. Miorin et al. (33) provided similar results, showing that the M58R mutation impairs ORF6 binding to the Nup98 and Rae1, abolishing its IFN antagonistic function. Additionally, the ORF6-D61L mutation was found to reduce interaction with Nup98 and Rae1, thereby weakening viral immune evasion (34).

NSP16 can modulate the host interferon response and directly promote inflammation during the infection process. NSP16-mediated methylation helps viral RNA evade recognition by PRRs (35). Banerjee et al. (27) found that NSP16 binds to the mRNA recognition domains of U1 and U2, blocking global mRNA splicing during SARS-CoV-2 infection and thereby inhibiting the interferon response to viral infection. Other studies have shown that NSP16 can upregulate hypoxia-inducible factor-1α expression, exacerbating IL-6 secretion (36). ORF7b also plays a critical role in viral pathogenesis. Research has shown that ORF7b induces host cell apoptosis through the tumor necrosis factor-α (TNF-α) pathway (37) and interacts with mitochondrial antiviral signaling protein (MAVS) to inhibit the RIG-I-like receptor signaling pathway, thereby suppressing innate immunity (38). Besides, ORF7b inhibits the phosphorylation of STAT1/2, thereby suppressing interferon expression (39). ORF10 can interact with several host proteins, including CUL2, ELOB, ELOC, MAP7D1, PPT1, RBX1, THTPA, TIMM8B, and ZYG11B, potentially contributing to the viral pathogenic process. Studies have shown that ORF10 can hijack CUL2ZYG11B, enhancing E3 ligase activity to degrade the intraflagellar transport complex B protein (IFT46), leading to ciliary dysfunction (40). ORF10 can induce mitophagy, degrading the mitochondrial MAVS, and subsequently inhibiting the expression of IFN-I (41). Notably, Liu et al. (42) used single-cell and bulk tissue transcriptomic data to investigate the characteristics and evolutionary differences in viral gene expression in SARS-CoV-2-infected cells. They found that ORF10 is highly expressed in infected cells from severe cases, while it is nearly undetectable in cells from moderate cases, suggesting a potential association with the pathogenesis of severe disease.

#### NSP10

On the one hand, NSP10 is a cofactor of the NSP16 methyltransferase, forming a complex with NSP16 to mediate viral RNA methylation (43). On the other hand, NSP10 can directly interact with NKRF, thereby mediating IL-8 expression, which may be related to the cytokine storm following viral infection (44).

Overall, although the aforementioned proteins of Class I play important roles in antiviral innate immune response and replication processes during viral infection, they have not undergone mutations in any of the five VOCs. This suggests that they may not be the primary factors driving the evolution of virus pathogenicity currently. However, it is also necessary to remain vigilant about the potential roles of mutations in these proteins in future variants.

### Class II (NSP6, NSP12, and N)

The Class II proteins exhibit some conserved mutations across multiple VOCs, suggesting that these mutations may enhance viral fitness. The impact of these proteins and their mutations on viral pathogenicity warrants further in-depth investigation.

#### NSP6

NSP6 is located in ORF1a and consists of 290 amino acids. It has been demonstrated that NSP6 is involved in viral replication, host cell autophagy, and inflammation, indicating it is a potential determinant of virus’s pathogenicity. The three-amino-acid deletion (SGF106-108 or LSG105-107) in NSP6 is conserved in multiple variants and may be related to the evolution of virus pathogenicity.

**Fig 2:**
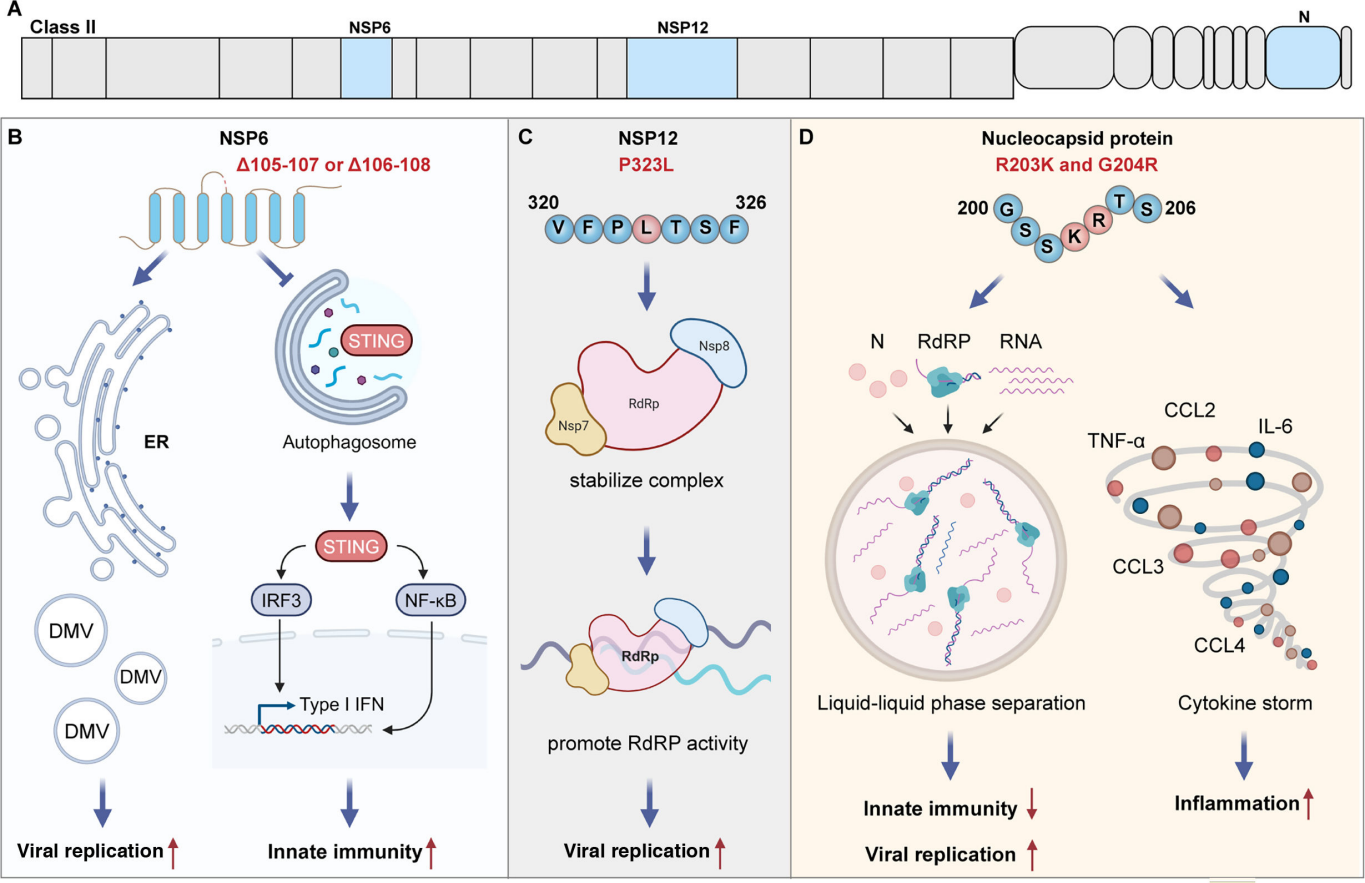
The major impacts of Class II protein mutations on the viral pathogenic process (**A)** The proteins of Class II. (**B)** The Δ105–107 or Δ106–108 in NSP6 enhances the production of double-membrane vesicles, thereby promoting viral replication. The three-amino-acid deletion also reduces the degradation of STING1, thereby enhancing the host’s innate immunity.** (C)** The NSP12-P323L mutation enhances the stability of the NSP12-NSP7-NSP8 complex, thereby promoting viral replication. (**D)** The R203K and G204R in N protein promote liquid-liquid phase separation, thereby enhancing viral replication and inhibiting innate immunity. Additionally, the R203K and G204R mutations are associated with an enhanced cytokine storm in infected patients.

One study found that overexpressed NSP6 can target HSPA5 in the endoplasmic reticulum, blocking the HSPA5-EIF2AK3 interaction and subsequently causing endoplasmic reticulum stress-induced autophagy (45). Autophagy induced by NSP6 further degrades the STING1 protein via lysosomes to inhibit interferon production, thereby promoting the immune evasion of SARS-CoV-2. Furthermore, the deletion of SGF106-108 or LSG105-107 in NSP6 reduces autophagy and STING1 degradation, thereby enhancing the host’s innate immune response, which may explain the attenuated virulence of variants such as Omicron (Fig. 2B) (45). However, other research found that the three-amino-acid deletion in NSP6 can enhance its antagonistic effect on IFN-I signaling and increase the virus’s virulence. Mice infected with the three-amino-acid deletion virus lost more weight than WA1-infected mice, and their survival rate was significantly lower. Further omics analysis revealed that the three-amino-acid deletion in NSP6 could mediate a more severe cytokine storm (46). Additionally, NSP6 can hijack ATP6AP1, blocking lysosomal acidification to inhibit autophagic flux, thereby activating NLRP3/ASC-dependent caspase-1 activation and triggering pyroptosis. The study also noted that the L37F mutation weakens the binding of NSP6 with ATP6AP1 and fails to mediate pyroptosis, which may be related to the virus’s attenuated virulence (47). In 2020, a study combining artificial intelligence, sequence alignment, and network analysis highlighted the importance of this site in the virus’s pathogenicity (48). However, L37F was lost in subsequent variants, suggesting it may not be a determinant in the evolution of viral pathogenicity.

In 2022, Ricciardi et al. (49) highlighted the critical role of NSP6 in viral replication, proposing that NSP6 is involved in the synthesis of replication organelles, while the ΔSGF mutation further enhances the NSP6 function (Fig. 2B). Then, in 2023, to identify the determinants of pathogenicity changes, Chen et al. (11) created a large panel of chimeric viruses, each incorporating the Omicron S protein along with one non-spike protein from Omicron, while the remaining proteins were derived from the WT virus. They discovered that mutations in the Omicron S protein alone did not significantly impact the virus’s replication capacity and pathogenicity. However, when they combined Omicron S with Omicron nsp6, they found that the mutations significantly reduced the virus’s replication and infection kinetics. Following the confirmation that NSP6 can reduce viral replication, Chen et al. (11) further evaluated the impact of mutations on viral pathogenicity. They found that the S/NSP6 mutations increased the 14-day survival rate of a susceptible mouse model from 0% to 71% after virus infection. Therefore, they suggested that NSP6 might be a key factor in altering SARS-CoV-2 pathogenicity. It is noteworthy that the increased survival rate in the susceptible mouse model was primarily due to the reduced viral replication kinetics. However, it was unclear whether the chimeric virus has lower pathogenicity at the same copies. Besides, the NSP6 mutations in Omicron-BA.1 include the ΔLSG105-107 and I189V, but I189V has been lost in subsequent Omicron subtypes. The three-amino-acid deletion has been present in multiple variants, including Alpha, Beta, and Gamma, and does not seem to correlate with the evolution trends of viral pathogenicity. Therefore, the NSP6 mutation alone is not sufficient to explain the evolution of viral pathogenicity. The synergistic interactions between NSP6 and other viral proteins suggest that NSP6 may influence the evolution of pathogenicity in an interdependent manner. As mentioned above, mutations in the S protein and NSP6 in the Omicron BA.1 variant reduce viral replication (11). NSP6 collaborates with NSP3 and NSP4 in the biogenesis of the replication organelle (49). Additionally, the ubiquitination of NSP6 and ORF7a promotes the activation of the NF-κB pathway (50). In summary, NSP6 has a significant impact on viral replication, inflammation, and pathogenicity. The combined effects of NSP6 mutations with those in other viral proteins, such as the Spike protein, NSP3, and NSP4, may play a critical role in the evolution of viral pathogenicity.

#### NSP12 (RdRP)

NSP12 is a core component of the RTC, with catalytic activity for viral RNA synthesis, making it a key protein influencing the viral replication process (51). The P323L mutation in NSP12 is highly conserved across the five VOCs, suggesting that it may enhance viral fitness. In 2023, Kim et al. (52) discovered that the P323L or P323L/G671S mutations stabilize the NSP12-NSP7-NSP8 complex and promote RdRP activity (Fig. 2C). Additionally, P323L or P323L/G671S mutations confer a replication advantage in the upper respiratory tract, thereby increasing the virus’s transmissibility. Apart from its role in viral replication, NSP12 also influences the host’s innate immune response. Wang et al. (53) found that NSP12 can inhibit the nuclear translocation of IRF3, thereby reducing IFN-I production, and this function is independent of RdRP activity. Furthermore, other studies have suggested that mutations in NSP12 may be related to viral pathogenicity. In 2020, Biswas et al. (54) analyzed the mutation profiles of SARS-CoV-2 isolated from mild and severe cases, finding that the P323L mutation in NSP12 was associated with disease severity. However, further experimental studies were still needed to determine whether P323L can directly impact disease severity. It is worth noting that, with the emergence of new variant strains, P323L has remained conserved in nearly all variants, which is inconsistent with the lower pathogenicity of subsequent strains. This raises the question of whether P323L truly impacts viral pathogenicity. Studies have shown that while introducing P323L into SARS-CoV-2 increases viral titers in the upper respiratory tract, it does not alter lung pathology or disease severity (54). Additionally, research suggests that P323L is a necessary mutation for the epidemiological success of SARS-CoV-2 variants, but no correlation has been observed between this mutation and COVID-19 mortality (55). Similarly, Goldswain et al. (56) found that P323L primarily functions in enhancing viral replication and transmission efficiency. Therefore, while mutations in NSP12 may be a determinant of viral fitness, their correlation with pathogenicity appears to be minimal.

#### Nucleocapsid protein

The nucleocapsid protein (N) is a structural protein of the SARS-CoV-2, consisting of 419 amino acids. The primary function of the N protein is to compact viral genomic RNA (gRNA), likely by facilitating the condensation of gRNA into ribonucleoprotein (RNP) complexes through phase separation. In addition, the N protein has been shown to be involved in viral RNA replication and inhibiting the host innate immune response. Numerous mutations in the N protein have been observed in VOCs. Among them, R203K and G204R mutations appearing in the Alpha, Gamma, and Omicron variants are potentially associated with viral fitness.

N protein regulates the host’s innate immune response and viral replication process through various mechanisms. In 2020, Savastano et al. (57) found that the N protein mediates intracellular liquid-liquid phase separation (LLPS), forming high-density protein/RNA complexes to enhance replication efficiency. These high-density protein/RNA condensates can recruit the RdRP complex, thereby facilitating efficient viral RNA transcription. In 2024, Ren et al. (58) discovered that the N protein can be SUMOylated by TRIM28, which then enhances LLPS, promoting the formation of viroplasms. These isolated biochemical reaction zones effectively facilitate viral replication and reduce the capture of viral components by the host immune system, thereby decreasing the innate immune response. The study also indicated that the R203K mutation augments the SUMOylation of NSP6, further boosting immune suppression. In contrast, the K65R mutation largely restores the IFN-β signaling (58). Moreover, N protein can directly inhibit the host cell’s innate immune response, aiding in viral replication during the early stages of infection. Stress granules (SGs) are cytoplasmic RNP condensates formed under stress conditions such as viral infections. Recent research has found that SGs are considered important platforms for activating the cGAS-STING and RIG-I/MDA5 signaling pathways. The binding of the N protein to SG nucleating proteins G3BP1/2 blocks SG formation, thereby inhibiting this antiviral strategy of the host. Besides, The N17A mutation disrupts the N-G3BP1 interaction, reducing viral replication *in vivo*, thus alleviating pathological damage (59–61).

Given the critical role of N protein in viral replication and immune evasion, numerous research groups have elucidated its impact on viral pathogenicity. Through a combination of *in silico* analyses and large-scale phylogenetic analyses, Wu et al. (62) discovered that the R203K and G204R mutations enhance viral adaptability and replication capacity. Further experimental results indicated that the viruses with R203K and G204R mutant exhibit increased infectivity in human lung cells and hamsters, causing more severe pathological damage. Similarly, Nagy et al. (63) found that the mutations G196V and S197L of N protein are associated with mild clinical outcomes, whereas R203K, G204R, and S194L are associated with more severe outcomes. Additionally, a research team from Saudi Arabia compared transcriptomic and proteomic data from nasopharyngeal swab samples of patients infected with wild-type virus and those infected with R203K and G204R mutant viruses. They found that cells from patients infected with the R203K and G204R mutant viruses exhibited significantly higher levels of pro-inflammatory cytokines and chemokines, indicating that these mutations may promote inflammation and are possibly associated with disease severity (Fig. 2D) (64). Consistent with these findings, Chen et al. (65) found that N protein itself can induce hyperinflammation. N protein can bind to intracellular Smad3 and downregulate CFTR expression in respiratory epithelial cells via microRNA 154, leading to intracellular chloride imbalance and mediating persistent inflammation.

Despite numerous studies illustrating the impact of the N protein on viral replication, immune evasion, and virulence, the mutation profile of the N protein does not seem to fully align with the trends in the evolution of viral pathogenicity. For instance, various studies have revealed the enhanced pathogenicity associated with the R203K and G204R mutations. However, the R203K and G204R mutations remain conserved across Omicron subvariants without significantly increasing the virus’s pathogenicity. This suggests that the reduced virulence of the Omicron variant is more likely attributed to mutations in other viral proteins, potentially overshadowing the influence of N protein mutations on viral pathogenicity. Specifically, the R203K and G204R mutations enhance viral replication and promote the release of inflammatory factors, thereby contributing to pathogenicity (62, 64, 66). However, mutations in the S protein and NSP6 of Omicron substantially reduce the virus’s replication capacity. Furthermore, mutations in the Spike protein attenuate fusogenicity, while the T492I mutation in NSP4 and the T9I mutation in the E protein mediate a lower inflammatory response. Indeed, clinical data indicate that, through the combined effects of these proteins, Omicron exhibits lower disease severity. Therefore, mutations in the N protein alone may not fully explain the evolution of viral pathogenicity. These findings further support that the pathogenicity of SARS-CoV-2 is not determined by a single protein but is jointly regulated by multiple virulence factors.

In general, various mutations in the Class II proteins are relatively conserved across the five VOCs, suggesting they may enhance viral fitness. However, these conserved mutations are inconsistent with the dynamic change of viral pathogenicity, indicating that these mutations alone cannot explain the mechanisms of viral pathogenicity evolution. Nonetheless, some conserved mutations do play a role in viral pathogenicity, as mutations in NSP6 and the spike protein in the Omicron variant significantly reduce viral titers in mice, thereby diminishing virulence.

### Class III (S)

The Class III proteins, including the S protein, exhibit a high mutation frequency, with different mutations present across the five VOCs. These high-frequency mutations may be related to selective pressures, thereby enhancing viral adaptability in various environments. Further analysis is needed to determine whether these mutations are associated with changes in pathogenicity. Here, we sought to investigate the correlation between S protein mutations and pathogenicity from the following perspectives: (i) the role of the S protein in the viral pathogenic process, (ii) the pathological damage induced by SARS-CoV-2 with S protein mutations in animal models, and (iii) the association between S protein mutations and clinical severity.

#### Spike protein

S protein is a structural protein of SARS-CoV-2 and assembles into homotrimers on the viral membrane. Each monomer of the S protein consists of two key subunits, S1 and S2. The S1 subunit contains the N-terminal domain (NTD) and the receptor-binding domain (RBD), which includes the receptor-binding motif that directly engages with the ACE2 receptor. The S2 subunit plays a critical role in facilitating the fusion of the viral and host cell membranes and contains the fusion peptide, two heptad repeats (HR1 and HR2), the transmembrane domain, and the cytoplasmic tail (67, 68). Multiple mutations in the S protein can affect its binding to ACE2, thereby modulating the infectivity of the virus. Zhang et al. (69) discovered that the D614G mutation increases viral infectivity by assembling more functional S protein into the virion. Tian et al. (70) indicated that the N501Y mutation in the S protein enhances the binding affinity between the RBD and ACE2, thereby increasing viral infectivity and transmission. A study combining computational and docking studies suggested that mutations such as Q493R, N501Y, S371L, S373P, S375F, Q498R, and T478K have a high binding affinity for human ACE2 (71).

**Fig 3:**
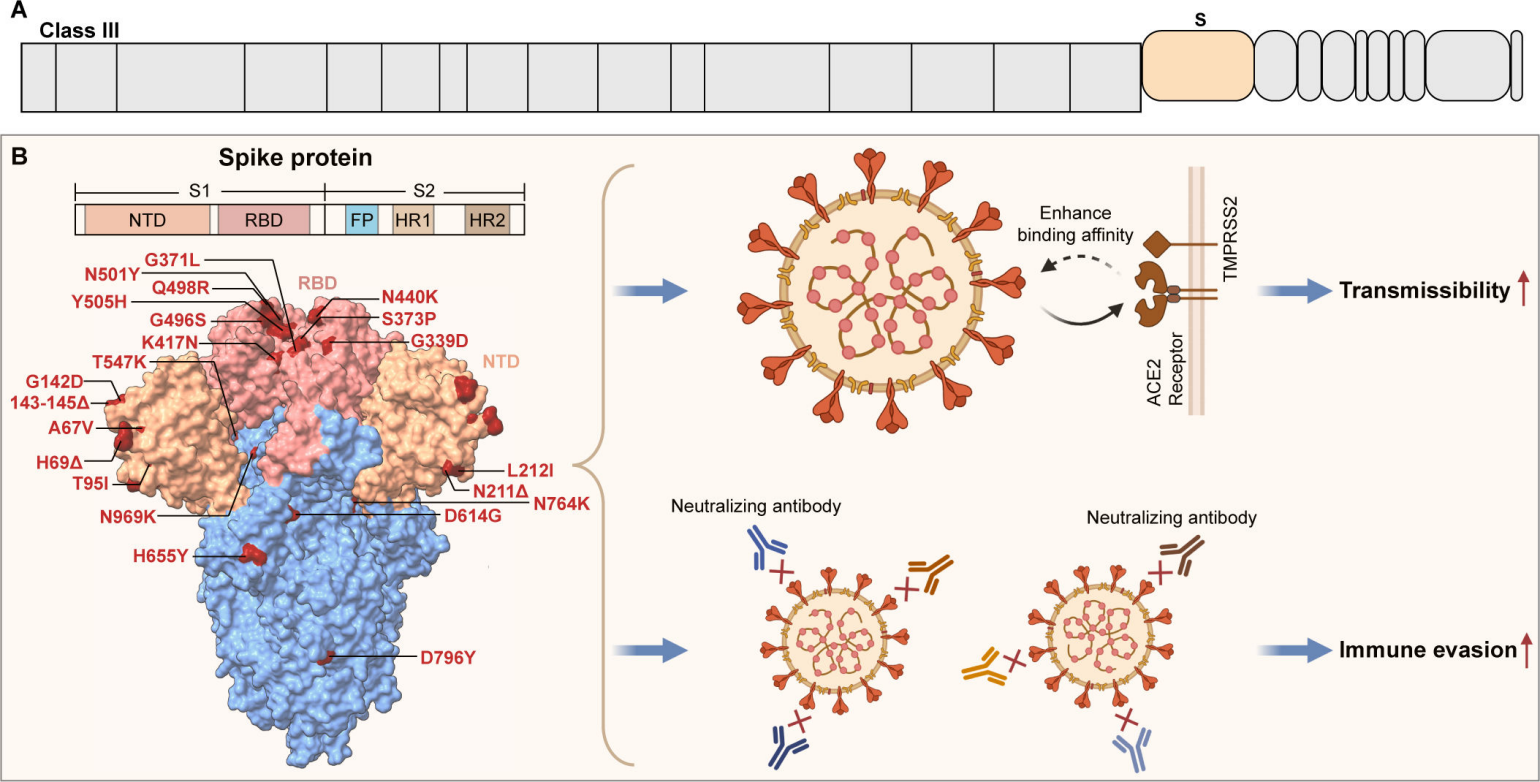
Spike protein mutations enhance the transmissibility and immune evasion capability of SARS-CoV-2. (**A)** The protein of Class III. (**B)** Left, a schematic diagram of the spike protein mutations in the Omicron variant. All mutation sites are highlighted in red within the structure. Right, the mutations in the Spike protein enhance the virus’s transmissibility and immune evasion capabilities. On the one hand, mutations in the spike protein can increase its binding affinity to receptors such as ACE2, promoting viral-cell fusion and thereby enhancing viral transmissibility. On the other hand, these mutations lead to antigenic drift, rendering neutralizing antibodies ineffective and increasing the virus’s immune evasion capability.

Furthermore, the surface of the S protein contains several neutralizing epitopes, which are the principal targets of neutralizing antibodies generated following infection or vaccination (67, 72). Therefore, the S protein is subject to high selective pressure, resulting in multiple mutations in VOCs that have increased viral transmissibility and the ability to evade vaccines and neutralizing antibodies (Fig. 3) (73, 74). A study identified over 100 monoclonal antibodies from individuals infected with SARS-CoV-2 and tested their neutralizing activity against mutant viruses. It was found that mutations in the S protein of the B.1.1.7 variant frequently conferred neutralization resistance to NTD-specific antibodies (75). Additionally, research by Upadhyay et al. (76) investigated the impact of 12 common mutations in the RBD of the spike protein on antibody escape potential. They found that the K417N, Y453F, N501Y, and K417T/E484K/N501Y reduced the affinity of the monoclonal antibody CC12.1, isolated from convalescent plasma of COVID-19 survivors. Besides, after isolating the Omicron variant from a patient, Planas et al. (77) tested the sensitivity of 9 monoclonal antibodies and 115 serum samples. They discovered that sera from various vaccinated individuals and convalescent patients had reduced or no neutralizing activity against Omicron. In 2023, further studies revealed that mutations such as R346T, N460K, K444T, V445P, and G446S in the BQ and XBB subvariants enhanced antibody escape, rendering monoclonal antibodies effective against the original Omicron variant largely ineffective against these new subvariants (78). Although these mutations in the S protein enhance antibody escape, facilitating the transmission and replication of mutant strains, there does not appear to be an increase in the pathogenicity of the current subvariants.

The role of the S protein in viral pathogenicity has also garnered attention. Several studies have found that the S protein can directly trigger inflammatory responses in host cells. In 2021, Li et al. (79) discovered that SARS-CoV-2 spike pseudovirions can upregulate intracellular ROS, inhibit the PI3K/AKT/mTOR signaling pathway, and thereby promote apoptosis and inflammatory responses. Likewise, Liang et al. (80) found that the S protein can inhibit mitophagy, upregulate ROS levels, and subsequently induce the expression of NLRP3 and IL-18 both *in vivo* and *in vitro*. Furthermore, S protein can activate the NF-κB pathway in a TLR-2-dependent manner, promoting the expression of IL-6, TNF-α, and IL-1β in cells or mice (81). The impact of S protein mutations on viral pathogenesis has been extensively studied. Kumar et al. (82) used structure-based computational assessments to evaluate the effects of S protein mutations on pathogenicity, identifying Y505H, N786K, T95I, N211I, N856K, and V213R as deleterious mutations. Additionally, one study indicated that the D614G mutation in the S protein has a higher incidence in severe patients compared to mild cases, suggesting that D614G might be related to viral pathogenicity (54). However, Volz et al. (83) analyzed 25,000 whole genome SARS-CoV-2 sequences and found no association between the D614G variant and higher mortality or clinical severity. In 2022, Saito et al. (9) discovered that the P681R mutation enhances the cleavage of the S protein, increasing viral fusogenicity, and virus carrying P681R exhibited higher pathogenicity compared to the WT virus. However, Furusawa’s results indicated that while P681R increased virus-cell fusion, it did not affect pathogenicity in hamsters (84). Similarly, Chen et al. (11) constructed chimeric viruses by introducing Omicron S protein mutations and compared their pathogenicity with the Omicron virus, finding that the S protein mutations alone did not reduce viral pathogenicity to the level observed in Omicron.

Altogether, S protein exhibits high-frequency mutations that play a significant role in enhancing viral adaptability. S protein plays a critical role in the virus’s ability to infect host cells, and its mutations are closely associated with changes in pathogenicity. However, the S protein mutations alone cannot fully account for the pathogenicity variations observed between these variants. In addition to the S protein, other factors and proteins also contribute to the pathogenicity of these variants. For instance, mutations in the S protein may work in concert with NSP6 mutations to reduce viral replication in the Omicron variant (11). In the Alpha, Beta, and Gamma variants, various accessory proteins, including ORF3a and ORF8, may act together with the S protein to contribute to viral pathogenesis (8). Using machine learning and comparative genomics, it has been found that, in addition to mutations in the Spike protein, enhancement of the nuclear localization signals in the nucleocapsid protein may also be associated with the virus’s high case fatality rate (85). Furthermore, mutations such as NSP4-T492I and E-T9I in the Omicron variant may contribute to its reduced pathogenicity (12, 14). Therefore, mutations in the Spike protein may influence viral pathogenicity in conjunction with multiple proteins, including NSP6, ORF3a, NSP4, N protein, and E protein.

### Class IV (NSP2, NSP3, NSP4, NSP5, NSP13, NSP14, ORF3a, E, M, ORF7a, and ORF8)

The Class IV proteins exhibit a small number of mutations in certain VOCs. Some of these proteins are proposed to be related to changes in viral pathogenicity. Investigating the roles of these proteins in the viral pathogenic process and understanding how their mutations affect viral pathogenicity is of significant importance.

#### NSP2

The N-terminus of NSP2 contains three zinc finger domains that can bind nucleic acids and regulate intracellular signaling pathways (86). Mass spectrometry results of NSP2 binding proteins indicate that NSP2 may be involved in several biological processes, such as endosome transport, protein translation, lipid synthesis, and modification (87, 88). Additionally, some studies found that NSP2 can activate NF-κB, promoting inflammatory responses (89). Laskar and Ali (90) compared the mutation profiles of SARS-CoV-2 from deceased and asymptomatic patients and found that certain mutations in NSP2 (D23Y, E66G, P181T, S211F, T371I, Q496P, and T85I) may enhance viral pathogenicity via supervised machine learning algorithms. The NSP2-T85I mutation might weaken the protein’s stability, thereby impacting pathogenesis (91). Other studies have indicated that mutations in NSP2, such as T224I, G262V, and T265I, may alter the pathogenic pathways of SARS-CoV-2 by disrupting protein or cellular signaling (92), despite these mutations were not conserved or retained in subsequent variants.

#### NSP3 (papain-like protease)

NSP3 is the largest protein of the NSPs, processing viral polyproteins to generate a functional replicase complex and playing a crucial role in viral replication and transcription (93). NSP3 has also been found to antagonize the host’s innate immune response. In 2020, Shin et al. (94) discovered that NSP3 cleaves the ubiquitin-like interferon-stimulated gene 15 protein (ISG15), thereby reducing IFN-I expression. Additionally, the pore formed by NSP3 and NSP4 on the double-membrane vesicles (DMVs) is crucial for the entry of viral RNA into the DMVs (95).

Mutations in NSP3 have potential impacts on viral replication and pathogenicity. Li et al. (96) isolated a strain with reduced virulence from model mice and found that the S676T mutation in NSP3 significantly decreased virus replication in cells and mice, thereby reducing virulence. Through multivariate logistic regression, Tan et al. (97) discovered that the K233Q and P78L mutations in NSP3 are associated with an increased risk of death. However, these potential key mutations have not appeared in the five VOCs, and no further studies elucidated the molecular mechanisms by which these mutations affect pathogenicity. Additionally, while inactivating mutations in NSP3 would obviously impair viral replication and thus reduce pathogenicity, such deleterious mutations are unlikely to be selectively retained in the virus.

#### NSP4

**Fig 4:**
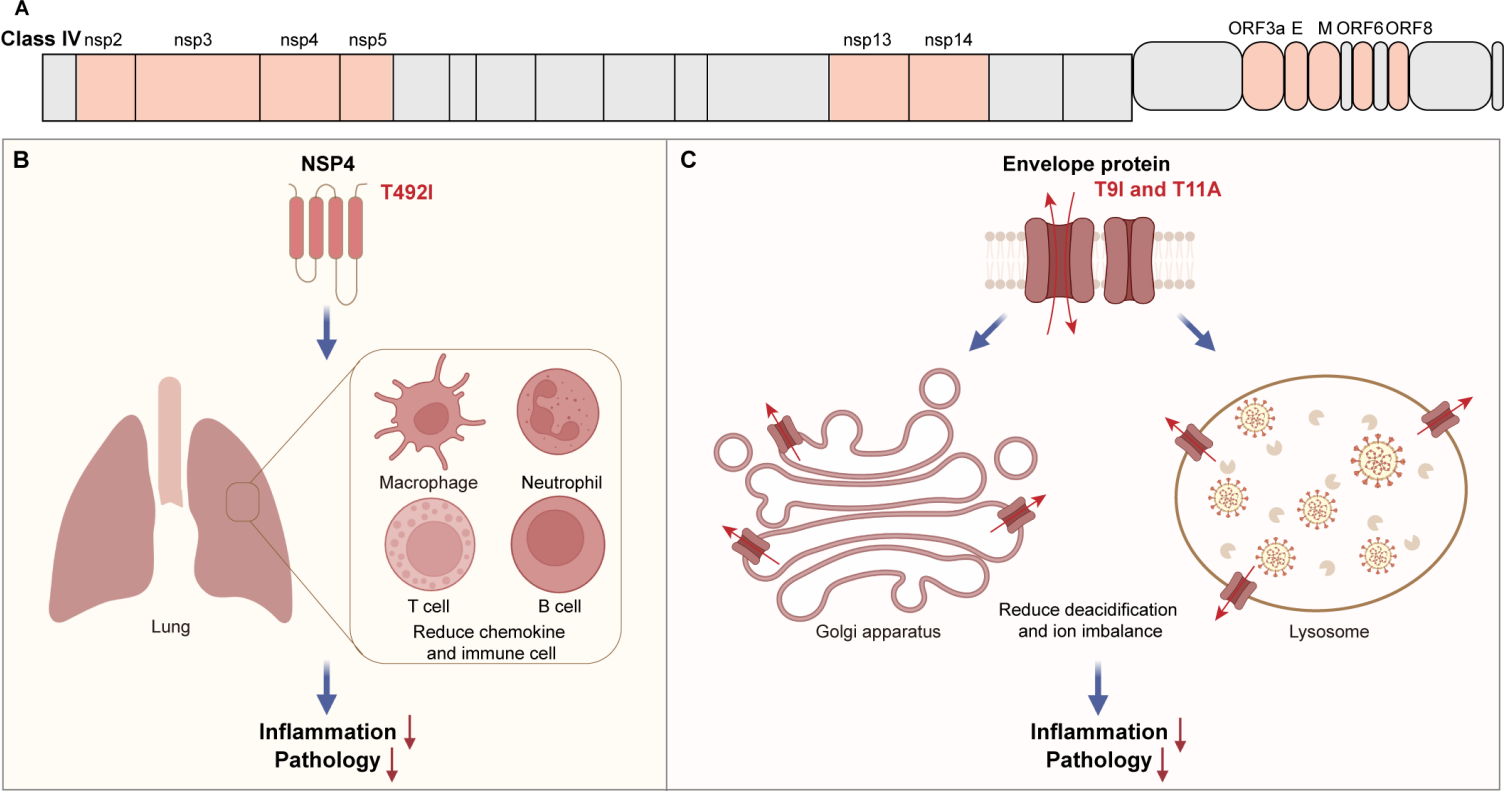
Mutations in NSP4 and E proteins reduce the pathogenicity of the virus. (**A)** The proteins of Class IV. (**B)** The NSP4-T492I mutation reduces the expression levels of chemokines post-infection, decreases the number of inflammatory cells in the lungs, and thereby reduces the pathogenicity of the virus. (**C)** The T9I and T11A mutations reduce the deacidification capability of the E channel, alleviate intracellular ion imbalance, and thereby mitigate inflammation and pathological damage.

NSP4 and NSP3 are involved in inducing the formation of DMVs during viral infection, playing a crucial role in viral replication (98). Additionally, NSP4 and NSP3 are minimal constituents in forming pores on DMVs, which are essential for viral RNA entry into DMVs (95). In 2023, Lin et al. (14) discovered that the T492I mutation in NSP4 in the Omicron variant enhances the binding between the enzyme and substrate, thereby increasing the cleavage efficiency of NSP5 and subsequently boosting the production of nonstructural proteins. Furthermore, the T492I mutation can suppress the production of chemokines in monocytes, which may contribute to the reduced pathogenicity of the Omicron variant (Fig. 4B). Notably, the T492I mutation is highly conserved among Omicron subvariants and aligns with the trend of lower pathogenicity, although it has also been observed in the Delta subvariant (21J) (14).

#### NSP5 (3CLpro), NSP13 (helicase), NSP14 (exonuclease), and ORF7a

Several proteins in Class IV can participate in the viral pathogenic process by inhibiting the host’s innate immune response. NSP5, also known as the main protease (Mpro or 3CLpro), plays a crucial role in processing virus-encoded polyproteins, making it an attractive drug target (99). NSP5 can antagonize interferon production by promoting the degradation of IRF3 (100). Research has shown that patients infected with SARS-CoV-2 strains carrying the NSP5-P108S mutation tend to experience a relatively milder clinical course compared to those infected with strains without this mutation (101). NSP13 inhibits the phosphorylation of TBK1, reducing the production of IFN3 (39). NSP13 and NSP14 can inhibit the nuclear translocation of IRF3 (28). Additionally, NSP14 possesses exoribonuclease and N7-methyltransferase activities, playing a crucial role in viral replication. Studies have demonstrated that NSP14 can inhibit host protein synthesis, and this function depends on its exoribonuclease and methyltransferase activities. Mutations such as H268A and D331A/G333A abolish its translational inhibition function (102). An analysis of 3,953 NSP14 variants, supplemented by computational predictions, identified mutations, including S418I and T524I as potential contributors to increased viral pathogenicity (103). ORF7a inhibits the phosphorylation of STAT2 (39). Additionally, Su et al. (104) found that ORF7a can activate NF-κB, thereby promoting the expression of proinflammatory cytokines. The correlation between mutations in ORF7a and changes in viral pathogenicity has drawn considerable attention. One study found that a 115-nucleotide deletion in ORF7a (ORF7a^Δ115^) increases the IFN response to SARS-CoV-2, leading to a growth defect (105). Sequencing and clinical data from a cohort of Romanian patients showed that the ORF7a-A105V mutation is associated with increased severity and mortality rates (106). Additionally, the H47Y mutation was found to weaken the immunosuppressive function of ORF7a, contributing to differences in viral pathogenesis (107). Overall, mutations in these proteins may influence viral pathogenicity by modulating the host’s innate immune response.

#### ORF3a

ORF3a is one of the accessory proteins encoded by SARS-CoV-2, consisting of 275 amino acids. Studies have found that ORF3a is localized to the plasma membrane, endoplasmic reticulum, and lysosomes. ORF3a is involved in viral replication, release, and the induction of host cell apoptosis and inflammation. ORF3a has various spontaneous mutations in the five VOCs, which may be related to the evolution of viral pathogenicity.

During SARS-CoV-2 infection, ORF3a plays various regulatory roles in the autophagy process, helping the virus evade the host surveillance mechanism (108). On the one hand, ORF3a binds to VSP39, a component of the HOPS complex, sequestering it in late lysosomes. This blocks the HOPS-mediated assembly of the STX17-SNAP29-VAMP8 SNARE complex, inhibiting the fusion of autophagosomes with late endosomes/lysosomes and the formation of autolysosomes (109). On the other hand, ORF3a disrupts the sorting of newly synthesized pro-cathepsin D to lysosomes (110). The inhibition of autophagy flux by ORF3a effectively helps the virus evade cellular surveillance mechanisms and suppresses innate immunity. In addition, ORF3a’s regulation of autophagy is also crucial for viral replication and release. ORF3a mediates the rearrangement of intracellular membranes, promoting the formation of autophagosomes, which leads to the accumulation of numerous membrane vesicles that form replication organelles for viral replication (30, 111). Furthermore, Chen et al. (112) discovered that SARS-CoV-2 ORF3a promotes the lysosomal targeting of the BORC-ARL8b complex, facilitating the movement of lysosomes toward the plasma membrane and exocytosis-related SNARE proteins. Overexpression of ORF3a significantly increases the release of the MHV-A59 coronavirus. The capacity of ORF3a to assist the virus in evading surveillance mechanisms, replication, and release indicates that it is critical for the viral pathogenic process.

In addition to regulating autophagy, ORF3a has also been confirmed to act as a virulence factor, mediating inflammasome activation and apoptosis. In 2022, Xu et al. (113) discovered that ORF3a can promote the assembly of the NLRP3 inflammasome by activating NF-κB or mediating intracellular potassium efflux, further leading to the upregulation of pro-inflammatory cytokine. Furthermore, ORF3a-mediated intracellular ion dysregulation can induce apoptosis. Using K^+^ channel inhibitors or introducing channel function loss mutations (C127S/C130S/C133S) can effectively reduce apoptosis levels (114, 115). One study found that knocking out ORF3a significantly reduces the expression levels of inflammatory cytokines in mice infected with SARS-CoV-2, further supporting its role as a virulence factor that is involved in the viral pathogenic process (8).

The above information suggests that ORF3a may be associated with changes in viral pathogenicity. Through computer analysis, a study compared the mutational profile of ORF3a with infection and mortality rates in over 20,000 COVID-19-positive cases spanning 23 countries. Researchers found a correlation between ORF3a mutations and higher mortality rates, identifying 13 deleterious mutations (Q57H, G251V, P25L, W149L, R126T, T176I, T217I, D142N, V90F, Y109C, D155Y, Y156N, and K67E) (116). In 2021, Nagy et al. (63) also pointed out that certain ORF3a mutations are associated with the severity of disease following SARS-CoV-2 infection. The G196V mutation is associated with mild symptoms, while the Q57H and G251V mutations are associated with patients in the ICU or with severe symptoms, and the S253P mutation is associated with fatal outcomes. Although these mutations may have played a key role in determining the pathogenicity of previous variants, they have all been lost in the Omicron subvariants. There are no ORF3a mutations in BA.1, while other subtypes such as BA.2 and BA.3 have only one new mutation, T223I, whose impact on viral pathogenicity remains unclear. In conclusion, ORF3a plays a crucial role in viral replication, immune evasion, and disease progression. Various ORF3a mutations have been associated with viral pathogenicity; however, these mutations have been lost in new variants and did not contribute to the subsequent evolution of viral virulence.

#### ORF8

ORF8a is one of the accessory proteins and has various effects on the host immune system. On the one hand, ORF8 can directly bind to the major histocompatibility complex class I and mediate its degradation via autophagy (117). Additionally, ORF8 inhibits the IFN-I signaling pathway, facilitating viral immune evasion (118). On the other hand, ORF8 can activate the IL-17 signaling pathway, promoting a cytokine storm in the host (119). The regulatory role of ORF8 on the host immune response suggests that it may influence the viral pathogenic process. Indeed, Bello-Perez et al. (120) found that viruses lacking ORF8 are associated with milder disease, indicating that ORF8 might contribute to SARS-CoV-2 virulence. However, McGrath et al. (121) found that the immune regulatory role of ORF8 on host cells appears to be beneficial, as mice infected with SARS-CoV-2 lacking ORF8 exhibited significant weight loss and increased macrophage infiltration in the lungs. Furthermore, this study also found that the spontaneous mutation E92K in ORF8 can exacerbate disease severity, suggesting that ORF8 might impact viral pathogenesis (121).

#### Membrane protein

The M protein defines the shape of the viral envelope and participates in the assembly of viral particles (122, 123). Additionally, the M protein can antagonize the host’s innate immune response. One study found that the M protein can bind TBK1 and induce TBK1 degradation via K48-linked ubiquitination, thereby interfering with the formation of the TRAF3–TANK–TBK1-IKKε complex and inhibiting IFN-I expression (124). Moreover, Zheng et al. (125) discovered that the M protein can target the RIG-I/MDA-5 signaling pathway, hindering the formation of the RIG-I, MAVS, TRAF3, and TBK1 complex, thereby suppressing the expression of IFN-I and IFN-III. Although a few mutations of the M protein have appeared in the Delta and Omicron variants, no studies have reported the impact of these mutations on viral pathogenicity.

#### Envelope protein

The envelope protein (E) is the smallest structural protein in SARS-CoV-2, consisting of 75 amino acids and containing a single transmembrane domain. Researches indicated that the E protein plays a crucial role in viral assembly and release. As a virulence factor, the E protein can mediate host cell death and inflammation, thereby playing a significant role in the viral pathogenic process.

E protein forms a cation channel and is permeable to various cations such as sodium, potassium, calcium, and magnesium (126). The transmembrane domain of the E protein forms a pentameric pore, which is regulated by proton and calcium (127, 128). Overexpression of the E protein can cause intracellular ion dysregulation, thereby triggering pyroptosis and severe inflammatory responses. Tail vein injection of purified E protein can induce ARDS-like damage in mice (126, 129). In 2021, Yalcinkaya et al. (130) treated macrophages with LPS and poly(I:C) to simulate the effects of more advanced infection, finding that overexpression of the E protein increased NLRP3 inflammasome activation in this setting. Additionally, E protein can directly activate TLR2, mediating inflammation and damage in the lungs of mice (131). In 2024, Xu et al. (132) further discovered that activation of TLR2 by the E protein increases intracellular Cl^−^ concentration ([Cl^−^]i) through upregulation of phosphodiesterase 4D expression in airway epithelial cells. Besides, E protein can downregulate the expression of tight junctional proteins, leading to airway epithelial barrier disruption and exacerbating pathological damage (132). The C-terminus of the E protein contains a PDZ-binding motif, which can bind to tight junction proteins ZO1 and PALS1, thereby disrupting the integrity of tight junction (133, 134). Subsequently, a study found that the E protein can mediate pyroptosis and release a large number of extracellular vesicles, aiding in viral immune evasion and transmission (129). Notably, inhibitors of E protein can effectively reduce viral titers in mice and alleviate lung damage, indicating that the E protein is a crucial factor in the viral pathogenic process (126). Similarly, Li et al. (135) found that the expression of the E protein is related to disease progression after viral infection. Their study showed that the E3 ligase RNF5 can mediate the ubiquitination and degradation of the E protein in the host, limiting viral replication. By comparing clinical data, Li et al. found that RNF5 expression is negatively correlated with disease severity.

A previous study found that T9I mutation alters the electrophysiological properties of E protein, thereby reducing its cytotoxicity and inflammation levels, suggesting a possible association with decreased viral pathogenicity (Fig. 4C) (12). Based on this, authors systematically analyzed spontaneous mutations in the E protein throughout the evolution of SARS-CoV-2, assessing the frequency changes, cytotoxicity, and pro-inflammatory capacity of the mutated proteins (13). The mutations can be categorized into three classes based on frequency changes and cytotoxicity. By comparing these classes with three independent clinical data sets, authors found a correlation between the frequency changes of E protein mutations and disease severity (13). The loss-of-function mutation T11A, which was previously identified, along with the T9I mutation, is present in the latest XBB variant (Fig. 4C). The double mutant protein (T9I/T11A) significantly reduced toxicity *in vivo* (13). These results further confirm that mutations in the E protein may be related to the evolution of SARS-CoV-2 pathogenicity. In the latest variants, including JN.1 and KP.2, the T9I mutation remains highly conserved across Omicron subvariants, consistent with the low pathogenicity of clinical data, suggesting that the E protein is critical for viral pathogenicity (136–138). In summary, E protein acts as a virulence factor, playing an important role in mediating host inflammation and disease progression. The mutations in the E protein are correlated with the current trends of viral pathogenicity, supporting the E protein as one of the major determinants of viral pathogenicity.

In summary, the Class IV proteins NSP2, NSP5, NSP13, NSP14, ORF7a, and M protein can affect host innate immunity and viral replication processes, thereby influencing viral pathogenicity. The T492I mutation in NSP4 and the T9I mutation in the E protein may be related to reduced viral pathogenicity. Notably, these two mutations are highly conserved across Omicron subvariants and are consistent with the trend of reduced viral pathogenicity.

## DISCUSSION

According to the hypothesis outlined above, the roles of viral virulence factors can be broadly classified into three categories. First, several virulence factors, including NSP2, NSP5, NSP6, NSP14, ORF7a, S protein, and N protein, enable the virus to evade host surveillance mechanisms, preventing clearance by the host immune system during the early stages of infection (39, 45, 46, 53, 118). Second, some virulence factors, such as NSP3, NSP4, NSP5, NSP6, NSP12, S protein, and N protein, promote viral replication or release, enabling the virus to proliferate extensively within the host (11, 49, 57, 95). Finally, certain virulence factors like NSP2, ORF3a, ORF8, S protein, N protein, and E protein act as initiators or promoters of inflammation and cell death, being highly expressed in the later stages of infection and further mediating cytokine storms (64, 65, 81, 89, 126). The roles of virulence factors in these three aspects are consistent with the clinical progression observed in severe patients. Clinical findings indicate that severe patients often manifest a delayed yet excessively active innate immune response, leading to a cytokine storm. The suppression or postponement of the host’s IFN-I-mediated innate immune response is a common tactic used by coronavirus, including SARS-CoV-1 and MERS-CoV, to increase their infectivity and sustain themselves in host tissues (139). Therefore, the pathogenicity of SARS-CoV-2 is likely determined by multiple virulence factors, including the virus’s ability to evade the immune system during early infection, its replication capability within host cells, and the toxicity of its pathogenic factor.

Based on current mutations and clinical data, the pathogenicity of each variant appears to be determined by multiple virulence factors, with no single viral protein fully explaining the mechanisms underlying the evolution of viral pathogenicity alone. (i) The proteins of Class I show no mutations in the five VOCs, suggesting they may have a minimal impact on the current evolution of viral pathogenicity. (ii) Among Class II proteins, the NSP6 mutations seem to affect viral replication only when present with specific S protein mutations (11, 49). The key mutations in the N protein and NSP12 are inconsistent with the trend of viral pathogenicity evolution (52, 64). (iii) In Class III, the mutations in the S protein directly affecting viral pathogenicity remain controversial, with differing clinical and experimental results (9, 54, 83, 84). (iv) As for Class IV, NSP2, NSP5, NSP13, NSP14, and ORF7a can influence the viral pathogenic process by interfering with host protein synthesis and innate immunity. The ORF8-E92K mutation, which might mediate higher viral pathogenicity, has disappeared in the Omicron variant. The NSP4-T492I and E-T9I mutations may be associated with reduced viral pathogenicity and are highly conserved across various Omicron subvariants. However, NSP4 and the E protein do not account for pathogenicity changes in the Alpha, Gamma, and Delta, as they are not mutated in these variants. This suggests that the alterations in pathogenicity observed in these variants are mediated by other viral proteins. The NSP4-T492I mutation, which appeared in the Delta subvariant 21J, does not align with the high pathogenicity of variants (14). Therefore, the evolution of SARS-CoV-2 pathogenicity is likely the result of the combined effects of these virulence factors. Based on mutations database and experimental and clinical findings, we hypothesize that certain proteins could play a significant role in the current evolution of viral pathogenicity. These proteins include NSP6, NSP12, N protein, S protein, NSP4, ORF3a, ORF8, and E (Table 2). The interplay among these proteins likely collectively determines the pathogenicity of variants. Therefore, in the future, it will be meaningful to develop a multidimensional computational model that incorporates the mechanisms, mutation profiles, and synergistic effects of these virulence factors to predict the pathogenicity of emerging variants. While viral pathogenicity is a multifaceted process, influenced not only by individual virulence factors but also by the complex interactions between various viral proteins, we are optimistic that future models will be better equipped to integrate these intricate protein interactions and provide more robust predictions. Our review aims to provide a systematic analysis of SARS-CoV-2 virulence factors, serving as a foundation that may assist in the development of these predictive models. In conclusion, the pathogenicity of SARS-CoV-2 is likely determined by multiple virulence factors. Analyzing the impact of different virulence factors and their mutations on the viral pathogenic process is of significant importance.

**TABLE 2:** Key mutations and effect of virulence factors

Class	Protein	Key mutations	Effect
Class II	NSP6	ΔSGF106-108/ΔLSG105-107	Attenuates pathogenicity (requires mutation of S)
		L37F	
	NSP12	P323L	Enhances pathogenicity (inconsistent with the trend)
	N protein	R203K	Enhances pathogenicity (inconsistent with the trend)
		G204R	
Class III	S protein	D614G	Enhances pathogenicity (controversial)
		P681R	
Class IV	NSP4	T492I	Attenuates pathogenicity
	ORF3a	Q57H	Enhances pathogenicity
		G251V	
	ORF8	E92K	Enhances pathogenicity
	E	T9I	Attenuates pathogenicity
		T11A	
